# A case of paediatric bladder bilharzioma in Johannesburg, South Africa

**DOI:** 10.1002/ccr3.2382

**Published:** 2019-08-21

**Authors:** Sohan Zane Pinto, Robin Friedman, Eunice Joy Van Den Berg

**Affiliations:** ^1^ Chris Hani Baragwanath Hospital University of Witwatersrand Johannesburg South Africa

**Keywords:** bilharzia, bilharzioma, bladder mass, hematuria, neglected tropical disease, schistosomiasis

## Abstract

On cystoscopy, a polypoidal tumor was observed and biopsied, and histology confirmed it to be an inflammatory mass with schistosoma eggs called a bilharzioma. We highlight this case to emphasize the silent destructive potential of schistosomiasis which the World Health Organization considers a Neglected Tropical Disease (NTD). A high degree of suspicion is often needed at the primary health care level to prevent morbidity.

## 1 INTRODUCTION

Schistosomiasis (also known as bilharzia) is a major cause of morbidity in South Africa with an estimated 4 million people infected.^1^ School‐age children are particularly vulnerable to this infection. The urogenital form of this Neglected Tropical Disease (NTD) caused by *Schistosoma haematobium* is linked to the increased transmission rate of HIV due to genital ulcers.^2^ Humans are the main hosts of the parasite. The adult worms are found in the pelvic venules from where they shed fertilized eggs into the tissues and lumens of pelvic organs. Mucosal erosions and chronic inflammation caused by these eggs can cause symptoms such as hematuria, dysuria, frequency, pelvic pain, intermenstrual bleeding, and vaginal discharge but can be asymptomatic especially in the early stages.^1^, ^3^ Female genital tract involvement can easily be mistaken for a sexually transmitted disease and, if not treated, it can result in infertility. Urinary tract involvement can result in a calcified or sclerosed poorly compliant bladder, ureteral stenosis, and renal failure. Sometimes the eggs get encapsulated in a fibrous granuloma or inflammatory pseudotumor called a bilharzioma that can manifest anywhere in the urogenital system.^4^ *S haematobium* is also one of the few helminths known to be a biological carcinogen due to its association with squamous cell carcinoma (SCC) of the bladder.^5^

Despite the serious morbidity and profound socio‐economic toll, schistosomiasis has on some of the most vulnerable sections of society in rural South Africa and neighboring countries, the disease is often overlooked owing to the prioritization of the big 3 diseases, namely HIV/AIDS, TB, and Malaria.^1^, ^2^ Great success has been achieved by integrating the HIV and TB prevention and treatment programs at primary care level. The integration of simple, inexpensive screening and treatment of schistosomiasis onto the already well‐established HIV program in endemic areas should be considered by health authorities.^2^, ^6^

This case report highlights the story of a relatively healthy‐looking child, from a rural background, harboring a hidden debilitating disease that was only picked up on cystoscopy. Multiple tests of urine microscopy for parasite eggs were unsurprisingly negative as it is known to have high specificity but low sensitivity.^7^ The primary healthcare center did not suspect bilharzia, and no empiric treatment was administered.

## 2 CASE REPORT

A 10‐year‐old girl with a 2‐year history of intermittent hematuria was referred to the Urology department of a tertiary hospital in Johannesburg. She had received multiple courses of antibiotics for the same at the local clinic. Urine microscopy done at the local clinics suggested urinary tract infections. No parasite eggs were seen on urine microscopy. The child described the hematuria to be terminal, with an initial clear urine stream followed by bloody urine toward the end of urination. This raised suspicion that the source of the bleed might be in the lower urinary tract. The mother gave history of travel to rural parts of the KwaZulu‐Natal province of South Africa and admitted that her child went for swims in the rivers.

**Figure 1:**
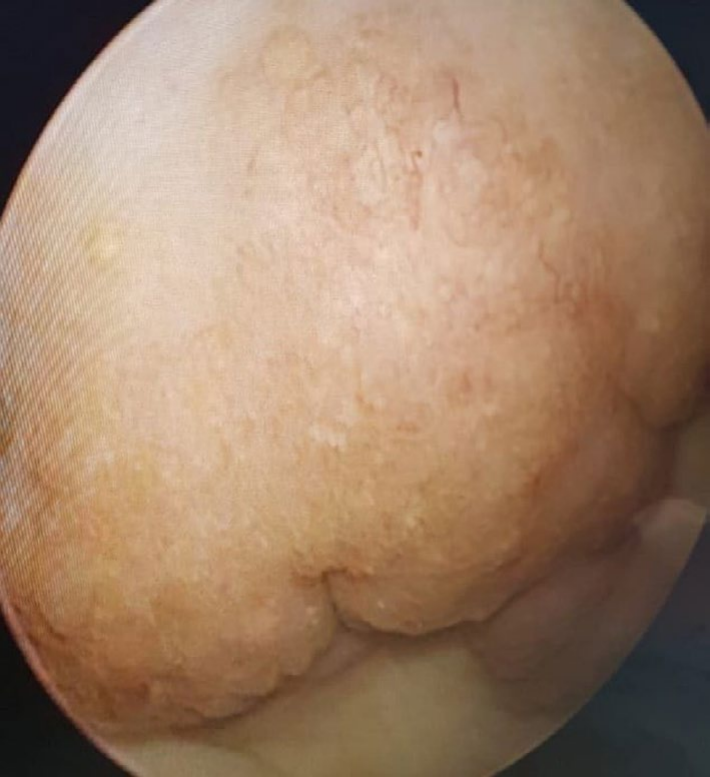
Cystoscopic view of the posterior bladder wall showing a polypoidal mass (bilharzioma) above and a pale granular mucosa below (sandy patches)

On examination, the child appeared clinically well and comfortable. No signs of malnutrition nor pallor. Systemic examination was unremarkable. A bedside ultrasound was done which showed normal kidneys, but a suspicious posterior wall bladder mass seen on ultrasound was concerning. Blood tests showed normal renal function and normal full blood count. The child was admitted for a cystoscopy under general anesthesia. On cystoscopy, multiple hemorrhagic polypoidal lesions were seen on the posterior wall of the bladder to the dome of the bladder (Figure 1). Adjacent bladder mucosa showed a granular pattern known as “sandy patches” which indicate areas of healed schistosomiasis.^8^ The ureteric orifices, trigone, and urethra were normal. Multiple biopsies were taken to rule out malignancy.

**Figure 2:**
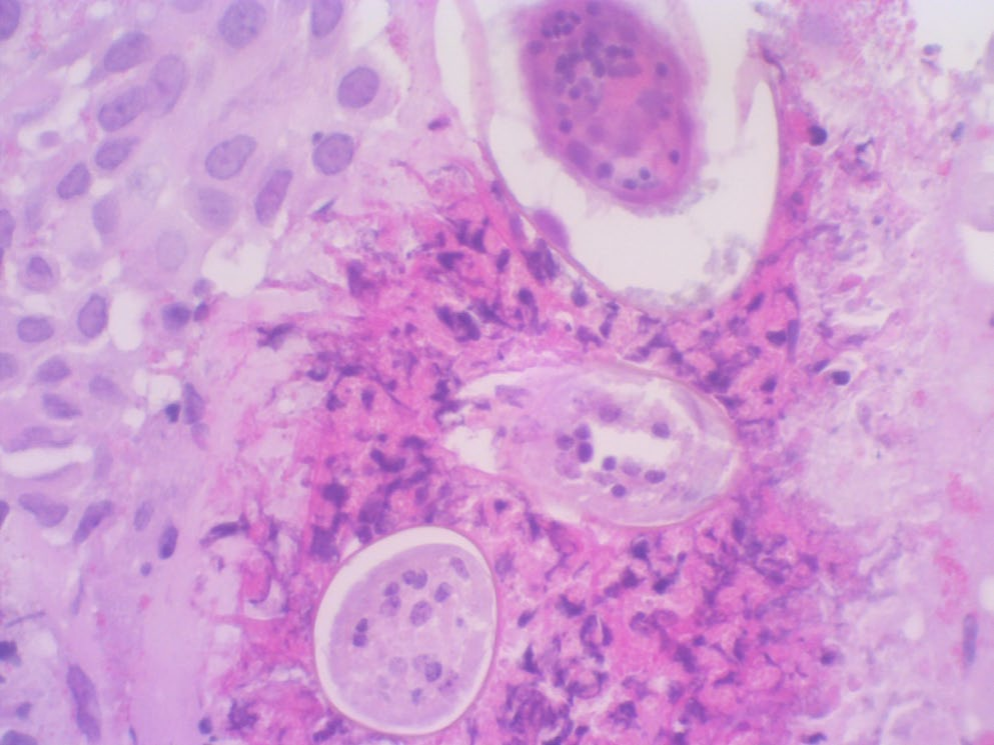
Histology slide from the bladder mass biopsy showing schistosomal ova and surrounding giant cell reaction

The histology revealed multiple viable Schistosoma ova on a background of dense inflammatory infiltrate, with a marked predominance of eosinophils (Figure 2). The child was treated with Praziquantel 40 mg/kg orally as a single dose.^1^ The child responded well to treatment and the hematuria resolved permanently. To rule out upper urogenital tract disease involvement, a CT intravenous pyelogram (IVP) was performed, the result of which was normal.^3^

At 6 months follow–up, the child was doing well with no complaints. A surveillance cystoscopy was performed which showed a normal bladder. Renal function tests and renal ultrasound were normal. The child was discharged after being counseled about warning signs of recurrence and preventive measures to avoid new contact with the pathogen.

## 3 DISCUSSION

Hematuria is the term to describe the presence of red blood cells in urine and can be microscopic or macroscopic. Macroscopic hematuria warrants urgent urological evaluation due to its higher association with urogenital malignancies. Urinary calculi, urogenital trauma, drugs, renal parenchymal disease, benign prostatic hyperplasia, and infective and noninfective urogenital inflammation are some of the other common causes of hematuria. A thorough history and examination often provides clues to the etiology. For example, initial hematuria suggests a urethral origin while terminal hematuria suggests a source at the bladder neck. Simple investigations such as urine dipstick, urine microscopy, and culture provide additional clues but do not dismiss the need for further urological evaluation. Cystoscopy for lower tract evaluation, ultrasound, and CTIVP for the upper tract usually completes the workup. A positive finding in the lower track on cystoscopy may still need upper tract evaluation to rule out synchronous lesions elsewhere.^9^

In the case described, the positive travel history to a bilharzia‐endemic area, the patient's longstanding symptoms for 2 years and her lack of exposure to bladder carcinogens like smoking and occupational chemicals reduced the chance of finding a malignancy. Nevertheless, the possibility of finding an infection like bilharzia should not be trivialized as the disease can cause permanent urological damage and even act as a risk factor for future malignancy.

**Figure 3:**
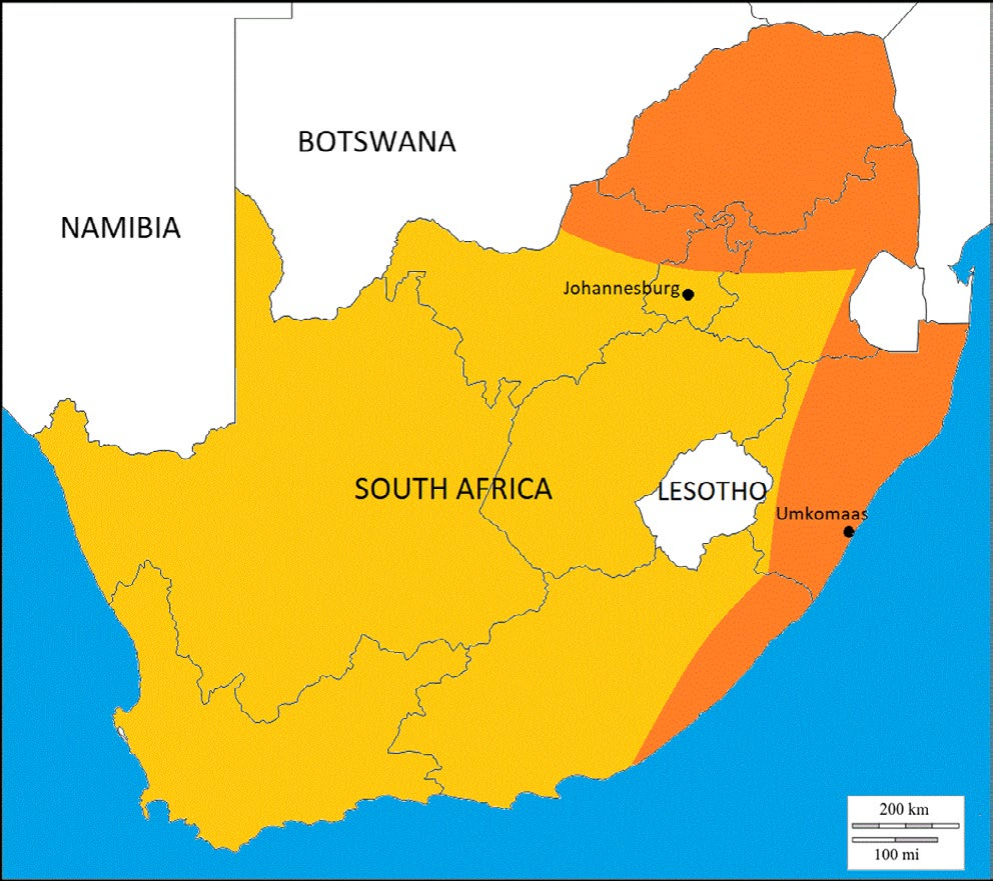
Map of South Africa with nonendemic areas in yellow and endemic areas in orange. The bilharzia‐endemic areas are found in the subtropical northern and eastern parts of the country. The patient who was being treated in nonendemic urban Johannesburg had a travel history which included swimming in the Umkomaas River on the rural east coast

Urinary schistosomiasis caused by the trematode (flatworm), *Schistosoma haematobium*, is endemic to Africa and the Middle East, especially in rural and agricultural areas. It is becoming more prevalent elsewhere with increasing rural‐urban migration and international travel with an estimated 90 million people infected worldwide.^3^, ^9^ Within South Africa, it is endemic to the east coast provinces (KwaZulu‐Natal and the Eastern cape) and the northern parts of the country (entire Limpopo province, northern parts of the North West, Gauteng and Mpumalanga provinces). These areas have a subtropical climate (Figure 3). Long, cold, and dry winters are associated with increased mortality of the snail intermediate host *Bulinus africanus*.^2^, ^10^

During the life cycle of *S haematobium,* the *Bulinus* snails act as an intermediate host. Infected human hosts shed eggs into freshwater bodies through urine and feces. Ciliated larvae from these eggs called miracidia infect the snails to form a sporocyst. Through asexual reproduction within the snail, a single sporocyst can produce up to 400 cercariae which are released back into the water bodies. Cercariae can penetrate the unbroken skin of a human host who comes in contact with infested fresh water. Humans act as the definitive host for the parasite's sexual reproduction phase. Once inside the human host, the cercariae shed their tails and transform into schistosomulae that migrate hematogenously to the lungs and then to the liver. The parasite now can mature into adult male and female worms which then preferentially migrate to the venous plexuses of the bladder and other pelvic organs. The adult worms can survive for up to 5 years during which they shed eggs into the pelvic venous circulation. A single female worm can lay up to a thousand ovoid eggs per day. The eggs cause most of the pathology in the host by stimulating an inflammatory response. To exit the host, the eggs must penetrate the endothelium and reach the lumen of the bladder. A smaller amount of eggs crosses the intestinal wall. The eggs are now released back into freshwater bodies via the urine or feces of infected individuals. On contact with fresh water, they hatch into miracidia and the cycle continues.^3^, ^9^, ^11^

The clinical manifestations of schistosomiasis are brought about by the inflammation caused by the parasite. It includes a transient acute phase when the cercariae penetrates human skin causing a pruritic maculopapular rash (swimmer's itch). This phase usually resolves when the traveler leaves the endemic area making future diagnosis difficult. A much rarer manifestation of the acute phase is called Katayama fever which is immune complex mediated and includes nonspecific symptoms such as fever, dry cough, fatigue, diarrhea, and headache and can be easily mistaken for a viral prodrome or even for malaria and enteric fever. The chronic phase is caused by the host's cell‐mediated immunity to the protracted oviposition and is commonly seen in endemic areas where residents are exposed to frequent reinfection and a high worm burden. The cell‐mediated response in the pelvic organs leads to granuloma formation, ulceration, fibrosis, calcifications and the complications thereof such as obstructive uropathy and malignancies. In the bladder, it can manifest as painless hematuria, dysuria, urinary frequency, or even urinary retention if the trigone is involved. Higher up in the ureters and kidneys fibrosis and granulomas can cause urinary obstruction (hydronephrosis) and renal failure. Over the years, chronic infection can become asymptomatic with low egg production leading to silent obstructive uropathy. Patients can even enter an asymptomatic chronic inactive phase where viable eggs are no longer found in urine or tissues, but permanent sequelae like poorly functioning or nonfunctioning kidneys are common. Involvement of female reproductive organs can lead to pelvic pain, dyspareunia, contact vaginal bleed, or vaginal discharge and are often misdiagnosed and mistreated as sexually transmitted diseases. Chronic fibrosis can lead to infertility. In male patients, it can manifest as hematospermia, testicular mass, testicular pain and infertility from seminal vesicle and ejaculatory duct involvement.^3^, ^4^, ^8^, ^11^

Diagnosis is by demonstration of the characteristic ova in urine which may be difficult in egg negative schistosomiasis like the patient described here. Maximal egg shedding occurs at noon so concentrating urine samples collected between 9am and 3pm should increase detection in low intensity infections. Hematuria and travel history should always warrant further investigation. Serological tests include enzyme linked immuno‐sorbent assays (ELISA) which are 90% sensitive and specific for *S haematobium* infection but cannot differentiate acute from chronic infections, and antibody titers remain positive even after curative chemotherapy. PCR or antigen detection in urine, serum, stool, and vaginal lavage samples is by far the most sensitive and specific tool to diagnose active infection but is relatively expensive for widespread use in the developing world. If clinical suspicion remains high and serology points to disease exposure, a tissue biopsy can be diagnostic. Clues on cystoscopy include the characteristic “sandy patches” which are healed schistosomiasis areas that are pale and granular. Active lesions may appear as patchy cystitis with surrounding fibrosis (cystitis cystica) or pseudo tubercles. The most worrisome finding on cystoscopy would be that of a fungating mass or malignant ulcer which could point to SCC. In female, genital schistosomiasis lesions on the vulva, vagina, and cervix may be sent for histology. On plain X‐ray, one might see linear calcifications of the urinary bladder. Ultrasound can pick up bladder wall thickening, bladder masses and hydronephrosis and hydroureter. CT scans can pick up smaller lesions in the upper urinary tract and colon.^8^, ^9^, ^11^

For treatment, the WHO recommends Praziquantel which has an efficacy of up to 90%. Side effects are rare and often mild and transient. A single stat oral dose of 40 mg/kg body weight (or divided into 2 doses given on the same day 8 hours apart) should suffice. It is advisable to monitor the patient posttreatment in terms of urine microscopy and bedside ultrasounds. Should there be any concerns of persistent infection, a repeat dose may be administered. Multiple doses of Praziquantel several weeks apart are recommended in areas of high transmission and reinfection. This is because Praziquantel is more efficacious against the adult worms of established infections than it is to the schistosomulae of early infections.^1^, ^3^, ^11^

Medical therapy alone can reverse early‐stage disease, and surgery should be reserved for emergency complications or persistent debilitating complications. The most common complication is obstructive uropathy from ureteral involvement causing hydronephrosis. Besides decompressing the obstructed kidneys with double‐J stents or nephrostomies, long‐term ureter repair procedures include endoscopic balloon dilatation, ureterouretostomy (end‐to‐end anastomosis of healthy ureter), or ureteric reimplantation into the bladder. Small contracted bladders can be augmented by procedures like hydrodistension and ileocystoplasty. Severely distorted urogenital anatomy may need permanent urinary diversion or permanent nephrostomies. A bladder SCC secondary to schistosomiasis will need a cystectomy.^11^

Children are especially vulnerable to intellectual and physical impairment as a result of chronic anemia, malnutrition, and sickness absenteeism. South Africa has endorsed resolution 19 of the 54th World Health Assembly which calls on member states to eliminate schistosomiasis. There are 3 points in the parasites life cycle where it can be targeted. The first is in the human host where the adult worm can live for years. This can be achieved by mass chemotherapy programs in endemic areas especially focusing on school‐age children. The second point is the parasites contact with water bodies. Improved sanitation and health education will not only prevent egg input into the environment but will also warn people to avoid contact with known infested water bodies. The third point is the snail where asexual reproduction allows the parasite to amplify rapidly. Snail control programs include mollusciciding, snail predators, or competitors.^1^, ^11^

### 3.1 Conclusion and teaching points

- Bilharzia is a neglected tropical disease (NTD) with a silent destructive potential. As human migration increases, healthcare personals must have a high degree of suspicion especially if there is reported travel to endemic areas.
- Urine microscopy to look for eggs is the most common inexpensive diagnostic tool used. However, as highlighted by the case above egg negative schistosomiasis is not uncommon.
- Any case of gross hematuria warrants further investigation of the urogenital track to rule out more sinister pathologies.
- Even when bilharzia is confirmed and treatment completed, follow‐up is necessary as some patients may require further doses.
- It is advisable for all patients to have a renal ultrasound to rule out bladder masses or obstructive uropathy. Any worrisome lesion should warrant further workup by a urologist.
- Health education and improved sanitation is imperative to prevent infections and reinfections.

## CONFLICT OF INTEREST

None declared.

## AUTHORS’ CONTRIBUTIONS

Sohan Zane Pinto: is the corresponding author, acquired and interpreted the data, acquired informed consent and assent, conducted literature review, wrote the manuscript, and approved the final manuscript. Robin Friedman: conceived and designed the study, and provided surgical expertise and senior supervision. Eunice Joy Van Den Berg: made high‐resolution picture of the histology slide and its description.
